# The Role of Metacognitive Strategy Monitoring and Control in the Relationship between Creative Mindsets and Divergent Thinking Performance

**DOI:** 10.3390/jintelligence10020035

**Published:** 2022-06-16

**Authors:** Xiaoyu Jia, Tianwei Xu, Yuchi Zhang

**Affiliations:** 1 College of Teacher Education, Southwest University, Chongqing 400715, China; jiaxiaoyu@126.com; 2 College of Science, Qiongtai Normal University, Haikou 571127, China; xtw77@163.com; 3 Key Laboratory of Child Cognition & Behavior Development of Hainan Province, Haikou 571127, China; 4 Department of Educational Technology, School of Wisdom Education, Jiangsu Normal University, Xuzhou 221116, China

**Keywords:** creative mindsets, metacognitive strategy monitoring and control, divergent thinking, self-strategic utility judgment, frequency of strategies usage

## Abstract

Previous research has shown that creative mindsets influence creativity. Compared with people with a fixed creative mindset, those with a growth creative mindset performed better in creative tasks. The underlying mechanism, however, is not completely understood. The present study has extended previous works to explore whether metacognitive strategy monitoring and control influence the relationship between creative mindsets and divergent thinking performance. The thinking aloud method was used to summarize four strategies in a divergent thinking task (an alternative uses task, AUT) in a pilot study: memory retrieval, splitting, property-based, and general use strategies. In the formal study, the creative mindsets scale, AUT, self-strategic utility judgment (i.e., an index of metacognitive strategy monitoring), and frequency of strategies usage (i.e., an index of metacognitive strategy control) were used to explore the relationships among creative mindsets, divergent thinking, and metacognitive strategy monitoring and control. The results indicated a positive correlation between a growth creative mindset and divergent thinking but a negative correlation between a fixed creative mindset and divergent thinking. More importantly, there were identified mediating roles of metacognitive monitoring and control of splitting and property-based strategies in the relationship between creative mindsets and divergent thinking. The findings reveal that creative mindsets are a critical predictor of divergent thinking, and metacognitive monitoring and control of abstract strategies mediate this association.

## 1. Introduction

Divergent thinking (DT), a key factor in creativity, refers to the generation of ideas that are both novel and useful for an open-ended problem (Guilford 1967). This can be reflected by a classic alternative uses task (AUT) that depends on the sum of the number of different ideas (fluency), the different cognitive categories of the ideas (flexibility), and the uncommonness of the ideas (originality). Studies have suggested that various task and individual factors can influence divergent thinking performance (Baas et al. 2011, 2015; Mehta et al. 2012). Among them, the role of individuals’ creative mindsets in creative thinking is a research hotspot (Hass et al. 2016; Jia et al. 2019; Karwowski 2014; O’Connor et al. 2013; Zhou et al. 2020).

### 1.1. Creative Mindsets and Divergent Thinking

Creative mindsets, within the implicit theory of creativity, refer to individuals’ beliefs about the fixed-versus-malleable nature of creativity (Karwowski 2014). It reflects the perceptions people hold about the nature of their creativity as something that is innate and unchangeable (fixed creative mindset) or growing and changeable (growth creative mindset). These two types of creative mindsets are relatively independent dimensions, rather than two opposite poles of the same continuum (Karwowski 2014; Karwowski et al. 2019). Studies have demonstrated the different consequences of creative mindsets on creative tasks (Hass et al. 2016; Karwowski 2014; O’Connor et al. 2013; Zhou et al. 2020; Royston and Reiter-Palmon 2017). For example, O’Connor et al. (2013) examined the relationship between creative mindsets and a series of creative tasks. Individuals’ creative mindsets were measured by a five-item Likert scale derived from Dweck’s (2006) implicit theories of intelligence scale, and creativity was reflected by self-perceptions of creativity, lifetime creative achievement, and AUT. The results revealed that people with a growth creative mindset performed better in these creative tasks. Similarly, several recent studies have found positive associations between growth creative mindset, creative self-concept (Hass et al. 2016), insight problem-solving efficiency (Royston and Reiter-Palmon 2017), and employees’ creativity in the workplace (Zhou et al. 2020).

Compared with people with a fixed creative mindset, those with a growth creative mindset perform better in creative tasks. However, the underlying mechanism is not completely understood. As there are complex divergent–convergent interactions with the construct of creativity (Eysenck 1995), the current study focused on exploring the relationship between creative mindsets and divergent thinking. Based on previous studies, we developed the following hypothesis:

**Hypothesis** **1.** *There is a relationship between creative mindsets and divergent thinking. Growth creative mindset will be positively related to divergent thinking performance, whereas fixed creative mindset will be negatively related to divergent thinking performance.*

### 1.2. Divergent Thinking and Metacognitive Strategy Monitoring and Control

Gilhooly et al. (2007) identified four types of strategies people adopted in AUTs by a think-aloud method. Specifically, memory use production was indicated by those uses retrieved from the episodic long-term memory system of individual people. Property use production referred to the retrieval of uses from the properties of the objects. Broad use-based production meant reviewing an object against a number of broad uses. Disassembly use production, however, involved the retrieval of uses by individuals from the components of the objects. It seems that this type of memory retrieval strategy appears to be relatively automatic, rapid, and shallow; and uses produced by it were common and lacking any personal–psychological creativity (Boden 2004), whereas other types of strategies tend to be slower, deeper, and more effortful; and uses produced by these were much more uncommon and novel. If people want to generate as many novel uses as possible, they should identify, select and switch to different strategies in the AUT process (Gilhooly et al. 2007). That is, individuals need to not only accurately monitor the novelty of uses produced by each strategy but also regulate the employment of their strategy, which reflects their top–down metacognitive strategy monitoring and control ability in AUT completion (Unsworth 2010).

According to metacognitive monitoring and control theory (Nelson and Narens 1994), individuals can regulate their learning behaviors, such as time allocation or strategy selection, based on their metacognitive monitoring judgment of ongoing cognitive activities (Dunlosky and Hertzog 2000; Koriat et al. 2006; Liu et al. 2004). For example, Wu and Liu (2006) examined children’s metacognitive monitoring and control ability during counting tasks. One of the judgments of utility about inversion strategy was used to reflect children’s metacognitive monitoring ability, whereas the inversion strategy selection was used to reflect their metacognitive control behavior. The results found a significant consistency between monitoring judgment and strategy selection, suggesting an interactive relationship between metacognitive strategy monitoring and control. In the current study, we explored whether this interactive relationship between metacognitive strategy monitoring and control exists in the AUT process. Given the significant role of metacognitive strategy monitoring and control in AUT (Unsworth 2010), we developed the following hypothesis:

**Hypothesis** **2.** *There is an interactive relationship between metacognitive strategy monitoring and control in the process of AUT. Individuals’ monitoring judgment of the utility of strategies in creativeness may affect their selection of strategies and, ultimately, AUT performance.*

### 1.3. Creative Mindsets, Divergent Thinking, and Metacognitive Strategy Monitoring and Control

Individuals with different types of mindsets make different cognitive strategy choices, especially in learning domains (Dweck 2006). Stipek and Gralinski (1996) explored relationships between fixed and growth mindsets, mastery and performance goal orientations, the use of deep or shallow strategies, and learning achievement. The causal model suggested that individuals with a fixed mindset tended to use more shallow strategies such as copying and memorization, whereas individuals with a growth mindset adopted deeper strategies such as paraphrasing and notetaking. Consequently, individuals with a growth mindset, rather than those with a fixed mindset, performed better in the learning tasks. The difference in strategy regulation between individuals with different types of mindsets was influenced by their different levels of metacognitive strategy monitoring abilities (Blackwell et al. 2007). A greater metacognitive strategy monitoring ability of individuals with a growth mindset led them to choose deeper strategies in the learning tasks.

Given the metacognitive strategy monitoring and control mechanism underlying AUT and the differences between different types of mindsets (Blackwell et al. 2007), we argued that such top–down metacognitive strategy monitoring and control processes may reveal how creative mindsets impact divergent thinking performance. Accordingly, we developed the following hypothesis:

**Hypothesis** **3.** *Individuals’ metacognitive strategy monitoring and control will play a mediating role in the relationship between creative mindsets and divergent thinking performance.*

To test these hypotheses, we firstly conducted a pilot study to identify the strategies involved in AUT by using a thinking aloud method (Green and Gilhooly 1996) in a Chinese setting. We then investigated the relationships among creative mindsets, metacognitive strategy monitoring and control, and AUT. The creative mindsets scale developed by Karwowski (2014) was used to reflect both fixed and growth creative mindsets that individuals endorsed simultaneously. Additionally, the self-strategic utility judgment, in which participants make a judgment regarding the novelty of ideas produced by each strategy, was used to reflect their metacognitive strategy monitoring ability. The frequency of strategy usage in AUT was used to reflect their metacognitive strategy control level. We hypothesized that compared with individuals with a fixed creative mindset, those with a growth creative mindset would rely on the accuracy of their metacognitive strategic monitoring and control to achieve better AUT performance.

## 2. Method

### 2.1. Participants

A power analysis (G*Power 3.1) was used to determine the minimum sample needed for an effect size of 0.26, alpha of 0.05, and power of 0.80, which is consistent with previous research on a similar topic (O’Connor et al. 2013). This resulted in an expected sample size of 87. Ninety university students participated in this study in exchange for CNY 10. Six participants did not complete the study and were thus excluded from further analyses. None of the 84 effective participants (17 males, *M* = 23.25, *SD* = 2.21) had previously participated in a similar study.

### 2.2. Materials 

#### 2.2.1. Creative Mindsets Scale (CMS)

The Chinese version of Karwowski’s (2014) 10-item creative mindsets scale translated by Zhou et al. (2020) was used to measure participants’ perceptions of the nature of creativity. Five items assess the extent to which one believes that creativity is fixed and unchangeable (e.g., “You have to be born a creator—without innate talent you can only be a scribbler”), and the other five test individuals’ belief that creativity is growing and changeable (e.g., “Everyone can create something great at some point if he or she is given appropriate conditions”). Participants were asked to rate these items on a 5-point scale ranging from 1 (strongly disagree) to 5 (strongly agree). The Cronbach’s α was 0.75 for fixed creative mindsets and 0.62 for growth creative mindsets in this study. Individuals’ fixed and growth creative mindsets were calculated by the sum of the corresponding five items, respectively. 

#### 2.2.2. Alternative Uses Task (AUT)

Participants were asked to complete an AUT to reflect their divergent thinking ability on a given answer sheet. Specifically, they were required to produce as many novel uses as possible for each of three common objects—carton, tire, and umbrella—in five minutes. Participants’ divergent thinking ability was measured by three different dimensions. Fluency was calculated by the number of responses given for the three objects. Flexibility was calculated by the number of categories given for the three objects. Originality was calculated by the averaged-response uniqueness for the three objects on a 5-point scale ranging from 1 (least original) to 5 (most original). These three dimensions were judged and averaged by three raters. Participants’ AUT score, an index to reflect their divergent thinking ability, was calculated by the mean score of standardized subscores for fluency, flexibility, and originality (Ivancovsky et al. 2018). 

In the pilot study, a thinking aloud method was used to summarize types of strategies involved in AUT. Specifically, another 31 participants (12 males, *M* = 20.35, *SD* = 1.87) took part in the pilot study. First, the experimenter demonstrated the meaning of “think aloud” to help participants understand it, taking the process of generating alternative uses of a newspaper as an example. Participants were then asked to think aloud as they worked on an AUT regarding as many unusual uses for five objects (i.e., carton, tire, umbrella, pencil, and brick) in 15 min. Their speech content was recorded with a digital recording pen. Next, the total of 465 min of think-aloud protocols for all participants were transcribed into 43,571 words, all of which were organized into several meaningful sentences. Three experts made codes for these sentences in two stages. In the initial coding, the experts identified seven processes inductively from early data: (1) Recalling: reporting a possible use by recalling from specific memory (e.g., “I remember in the movies they used bricks as a killing tool”). (2) Object-splitting: reporting a possible use by splitting objects into several parts (e.g., “Take the umbrella apart so that the top can be used to make clothes, bags”). (3) Repeating: repeating an already stated use (e.g., “a killing tool, tool”). (4) Object-naming: repeating the name of an object (e.g., “e, umbrella, umbrella, umbrella”). (5) General use-retrieving: reporting the general or common use of an object (e.g., “The pencil can be used to write, draw”). (6) Property retrieval: reporting the properties of an object (e.g., “The pencil is sharp”). (7) Impasse: inability to report any further uses (e.g., “I don’t know any other uses”). In the generic coding, experts grouped these initial codes into four larger strategies: (1) A memory retrieval strategy was indicated by those novel uses retrieved from the memory system covering the direct and indirect experiences of the self, such as “umbrella was used as a tool for taking photos in my childhood”. (2) A splitting strategy was indicated when participants split and used parts of objects to generate novel uses, such as “I can split the umbrella fabric and make it into a bag”. (3) A property-based strategy was indicated by those uses generated by the properties of objects such as size, shape, color, and texture, such as “the long handle of umbrella can be used as a walking stick”. (4) A general use strategy was identified through participants’ retrieval of general or broad uses of the objects, such as “the pencil can be used to write or draw”. To sum up, there were four kinds of strategies identified in the AUT, namely, memory retrieval strategy, splitting strategy, property-based strategy and general use strategy.

#### 2.2.3. Self-Strategic Utility Judgment (Utility Judgment)

In this questionnaire, participants were provided with definitions and examples of the four strategies about memory retrieval, splitting, property-based, and general use involved in AUT, to ensure their understanding. Participants were then asked to evaluate the degree of utility of the four strategies in generating novel uses in AUT on a 5-point scale ranging from 1 (least effective) to 5 (most effective), respectively. In other words, each strategy received a utility judgment value. This self-strategic utility judgment was an index of an individual’s metacognitive strategy monitoring ability (Dunlosky and Hertzog 2000; Liu et al. 2004). 

#### 2.2.4. Frequency of Strategies Usage (Usage Frequency)

After participants completed the self-strategic utility judgment, each strategy was labeled numerically such that participants could insert the corresponding strategy number behind each answer (1 = memory retrieval strategy; 2 = splitting strategy; 3 = property-based strategy; 4 = general use strategy). As in Wu and Liu (2006), this frequency of strategies usage was regarded as a reflection of the individual’s metacognitive strategy control ability. The usage frequency for each strategy was the average number for the three objects in this study. 

### 2.3. Procedure

Four types of strategies involved in AUT were summarized in the pilot study. In the formal study, participants were asked to complete the creative mindset scale, AUT, utility judgment, and usage frequency reactions in turn. It is worth noting that half of the participants first completed the creative mindset scale and then the AUT, while the others did the opposite to balance the order effect. 

### 2.4. Data Analysis

The purpose of this study was to explore the roles of metacognitive monitoring and control ability of the four different kinds of strategies in the relationship between creative mindsets and AUT performance, respectively. Accordingly, descriptive statistics and correlation analyses were tested, firstly using SPSS 23.0. The individual variables referred to creative mindset (growth or fixed), AUT score, utility judgment and usage frequency of each of the four strategies. Secondly, multiple mediation analyses were conducted using Hayes macro PROCESS in SPSS 23.0. The assignation of growth or fixed creative mindset was an independent variable; utility judgment and usage frequency for each of the four strategies were mediating variables; and AUT score was an outcome variable. Therefore, 8 models were conducted in the multiple mediation analyses. A total of 5000 bootstrap samples were used, and if the 95% confidence interval (CI) did not include 0, the mediating effect was significant. 

## 3. Results

Overall, the 84 participants answered the CMS, the AUT, and the self-strategic utility judgment comprehensively. The N of frequency of the usage of the four strategies was different because not all 84 participants used any of the four strategies at once. Specifically, for memory retrieval strategy, *N* = 83; for splitting retrieval strategy, *N* = 83; for property-based strategy, *N* = 84; and for the general used strategy, *N* = 81. Nevertheless, after careful consideration, we included the data of all 84 participants in the following correlation and multiple mediation analyses. 

### 3.1. The Correlations among Variables 

The means, standard deviations, and correlations among variables are presented in Table 1. Growth and fixed creative mindsets were negatively correlated. Growth creative mindset was positively correlated with AUT score, whereas fixed creative mindset was negatively correlated. Moreover, the growth creative mindset was positively correlated with utility judgment of the splitting strategy, and with usage frequency of both splitting and property-based strategies. The fixed creative mindset, however, was only negatively correlated with utility judgment of the splitting strategy. Additionally, AUT score was positively correlated with both utility judgment and usage frequency of the four kinds of strategies, except for the utility judgment of the general use strategy. Together, the above relationships provide foundations for further examination of the proposed mediation pathways.

### 3.2. The Mediating Effect of Metacognitive Strategy Monitoring and Control on the Relationship between Creative Mindsets and Divergent Thinking

**Figure 1:**
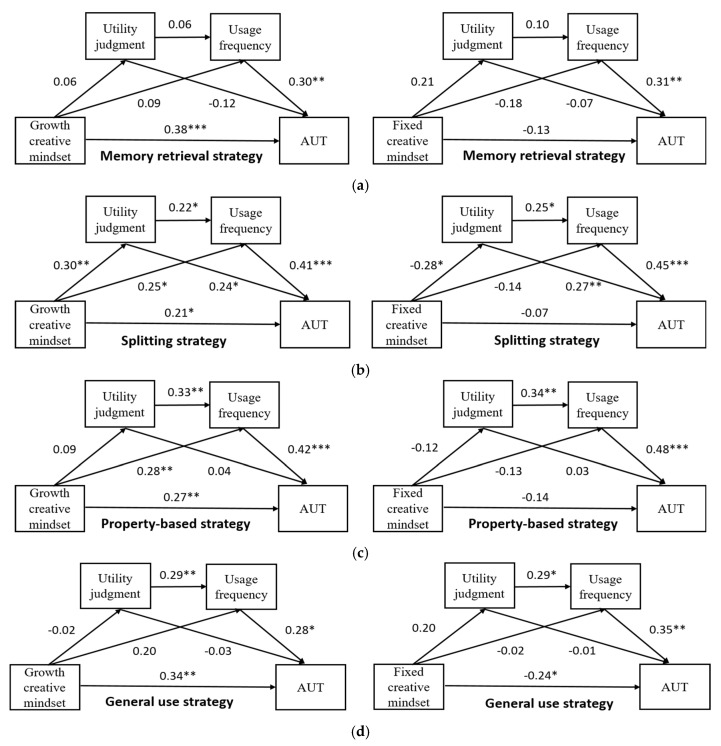
Sequential mediation model of growth/fixed creative mindset as predictor of creativity mediated by metacognitive Strategy monitoring and control for four different strategies. Standardized regression coefficients are displayed for all paths. Figures (**a**–**d**) represent memory retrieval strategy, splitting strategy, property-based strategy, and general use strategy, respectively. * *p* < 0.05; ** *p* < 0.01; *** *p* < 0.001.

Multiple mediation analyses were conducted using Hayes macro PROCESS in SPSS to assess the role of metacognitive strategy monitoring (reflected by utility judgment) and control (reflected by usage frequency) in the relationship between creative mindsets (growth or fixed) and divergent thinking (reflected by the AUT score). The total mediating effect comprised creative mindsets through utility judgment (path 1), usage frequency (path 2), and a serial mediation of utility judgment and usage frequency (path 3). The results of the sequential mediation analysis of the growth and fixed creative mindsets for the four different strategies are shown in Figure 1a–d, respectively: 

(1) For the memory retrieval strategy, the 95% CIs corresponding to the three paths were [−0.05, 0.02], [−0.04, 0.10], [−0.005, 0.01] for growth creative mindset and [−0.08, 0.04], [−0.13, 0.01], [−0.004, 0.03] for fixed creative mindset. This indicates that the mediating effects of the three paths were not significant for both creative mindsets. (2) For the splitting retrieval strategy, the 95% CIs corresponding to the three paths were [0.007, 0.15], [0.02, 0.19], [0.004, 0.06] for growth creative mindset and [−0.17, −0.004], [−0.15, 0.04], [−0.07, −0.002] for fixed creative mindset, indicating that the mediation effects via usage frequency alone were only significant for growth but not fixed creative mindset. Meanwhile, the mediation effects via utility judgment and utility judgment on usage frequency were significant for both creative mindsets. (3) For the property-based strategy, the 95% CIs corresponding to the three paths were [−0.02, 0.04], [0.04, 0.21], [−0.01, 0.04] for growth creative mindset and [−0.05, 0.03], [−0.18, 0.03], [−0.07, 0.03] for fixed creative mindset, indicating that only the mediation effect via usage frequency was significant for growth creative mindset. (4) For the general use strategy, the 95% CIs corresponding to the three paths were [−0.03, 0.03], [−0.001, 0.16], [−0.02, 0.02] and [−0.06, 0.05], [−0.08, 0.06], [−0.004, 0.07], indicating that the mediating effects of the three paths were not significant for both creative mindsets.

## 4. Discussion and Conclusions

Studies have found that individuals’ mindsets influence their performance in various domains, such as learning, music, and morality (Chiu et al. 1997; Claro et al. 2016; Daniel et al. 2015; Paunesku et al. 2015). In this study, we investigated whether there was a relationship between individuals’ creative mindsets and divergent thinking, and we examined the mediating role of metacognitive strategy monitoring and control in this association. The results revealed a positive correlation between growth creative mindset and divergent thinking, whereas a negative correlation between fixed creative mindset and divergent thinking was identified. More importantly, metacognitive monitoring and control of the splitting and property-based strategies played a mediating role in these associations. 

Recent research has highlighted the role of idea generation strategies in AUT (Beaty and Silvia 2012; Gilhooly et al. 2007). Semantic analysis of thinking aloud protocols in our study revealed that there are four strategies used by individuals in AUT: memory retrieval, splitting, property-based, and general use strategies. Differing from the broad use-based strategy in Gilhooly et al. (2007), the general use strategy was here defined as the retrieval by individuals of general and regular uses of the objects. This general use strategy, similar to memory retrieval strategy, appears to be relatively automatic and shallow and uses produced by it were common and regular, whereas splitting and property-based strategies tended to be slower, deeper, and more effortful, and uses produced by them were much more uncommon and novel (Beaty and Silvia 2012; Boden 2004). From this point of view, it is worth noting that individuals’ accurate metacognitive monitoring abilities for splitting and property-based strategies were reflected by higher utility judgment values, whereas their accurate metacognitive monitoring abilities for memory and general use strategies were reflected by lower utility judgment values on the 5-point scale ranging from 1 (least effective) to 5 (most effective). In the AUT process, individuals should consciously overcome the interference of common ideas easily produced by shallow strategies and deliberately switch to much more deep strategies to increase the possibility of novel idea generation (Beaty and Silvia 2012), which involves the activation of the top–down metacognitive strategic monitoring and control system. 

As is consistent with previous research (Karwowski 2014; Karwowski et al. 2019; Zhou et al. 2020), our study found that holding a growth creative mindset boosts AUT performance, while a fixed creative mindset hinders AUT performance. This critical AUT difference between individuals with a growth and those with a fixed creative mindset lies in their relative metacognitive strategy monitoring and control abilities. Studies of the relationship between mindsets and strategies in the learning domain (Braten and Olaussen 1998; Dupeyrat and Mariné 2005; Stipek and Gralinski 1996) have suggested that individuals with a growth mindset tend to use much more deeply cognitive strategies (Dahl et al. 2005) to perform better in the learning tasks. In the present study, we found significantly positive correlations between growth creative mindset and frequency usage of splitting and property-based strategies. Considering that uses produced by splitting and property-based strategies are more likely to be novel and creative (Beaty and Silvia 2012), the greater use of these two strategies for individuals with growth mindset might partly explain their better AUT performance. Furthermore, we also found that the higher frequency of splitting strategy employed by the aforementioned individuals with growth creative mindset was based on their accurate utility judgment for it, reflecting a positive interaction of metacognitive monitoring-control ability of splitting strategy for individuals with growth creative mindset. That is to say, compared with individuals with fixed creative mindset, those with growth creative mindset gave a higher utility judgment for splitting strategy and used it more frequently to gain a better performance in AUT. This higher metacognitive monitoring and control ability of individuals with growth mindset has been explored by empirical and neurophysiological studies (Blackwell et al. 2007; Ehrlinger et al. 2016; Metcalfe and Finn 2008). For example, an event-related potential (ERP) study by Mangels et al. (2006) found that the anterior cingulate gyrus (ACC), a core area of the metacognitive monitoring and control system, was activated in the process of completing challenging academic tasks in individuals with a growth mindset (Ridderinkhof et al. 2004). Over the course of AUT, we found that the metacognitive monitoring and control of splitting and property-based strategies, more than memory retrieval and general use strategies, played mediated roles in the relationship between creative mindsets and AUT performance. The possible reason was that there are differences in the activation of metacognitive monitoring-control for different strategies (Beaty and Silvia 2012). Specifically, memory retrieval and general use strategies were regarded as more dependent on automatic spreading activation than effortful executive functioning, whereas the splitting and property-based strategies were based on more abstract semantic knowledge of objects’ properties. From this point of view, the activation of top–down metacognitive monitoring-control ability was stronger among individuals adopting splitting and property-used strategies in AUT. Accordingly, this higher ability of metacognitive monitoring and control of splitting and property-based strategies for individuals with a growth creative mindset contributed to their better AUT performance. 

To summarize, the current study contributes to research on the relationships between creative mindsets, metacognition, and divergent thinking. However, it has several limitations. First, the measurement of strategies usage was retrospective, as we gave participants four types of strategies to match their answers, rather than letting them self-analyze strategies in the AUT process. Although the provided strategy types summarized in the pilot study might have reflected the actual strategies’ analysis of participants in the formal study, the much more valid measurements of participants’ self-analyzed strategies while completing AUT should be considered in future studies. Second, considering that creative mindsets have strong positive relationships with other creative self-concepts, such as creative self-efficacy and creative personal identity (Karwowski 2014; Hass et al. 2016), the role of creative mindsets in AUT might be partly influenced by these related variables. Further research is essential to explore the influence of creative mindsets on creativity by exploring how these mindsets interact with related variables. Third, we must acknowledge the within–between confounding effect given the cross-sectional design (Hsu et al. 2022) used in the current study; that is, we could not conclude whether this relationships among creative mindsets, metacognitive strategies monitoring and control, and creative thinking were due to associations at the within– or between–person levels. Future developments in experimental design are needed to better explore this issue. Fourth, although some ambiguous items of CMS have been clearly described by experts from a Chinese context regardingthe procedure of English–Chinese translation, the CMS should nevertheless be used with caution in the future. Additionally, although the order of AUT and creative mindset measurements was counterbalanced, the effect of interaction between the two measures could not be excluded because of the state component in creativity and creative mindset. A counterbalanced order with a one-week interval could be a possible solution in future research. Finally, the practical implications of fostering individuals’ divergent thinking from the perspective of creative mindset intervention, apart from individuals’ metacognitive skills teaching (Hargrove and Nietfeld 2015), could be considered in the future. Specifically, previous studies have examined the effects of general mindset intervention in many disciplines such as learning, writing, anxiety, and musicality (Paunesku et al. 2015; Schleider and Weisz 2018). The improvement of divergent thinking through creative mindset intervention shows promise, because of their close relationship between each other.

## Figures and Tables

**Table 1:** Means (M), standard deviations (SD), and correlations among variables.

	Variables	*M*	*SD*	1	2	3	4	5	6	7	8	9	10	11
1	Growth	17.64	3.54	1	−0.33 *	0.41 **	0.06	0.08	0.30 **	0.33 **	0.09	0.31 **	−0.05	0.19
2	Fixed	10.62	3.82			−0.22 *	0.21	−0.16	−0.28 *	−0.20	−0.12	−0.17	0.20	0.03
3	AUT	0.00	2.30				−0.08	0.34 **	0.42 **	0.57 **	0.22 *	0.52 **	0.02	0.35 **
4	S1UJ	2.98	0.96					0.06	−0.04	−0.06	0.11	−0.17	0.53 **	0.05
5	S1UF	9.72	4.65						0.005	0.07	−0.14	−0.18	0.02	0.14
6	S2UJ	3.53	1.04							0.29 **	0.15	0.33 **	−0.12	0.16
7	S2UF	4.20	3.48								0.25 *	0.33 **	0.12	0.19
8	S3UJ	3.46	0.90									0.36 **	0.12	−0.03
9	S3UF	9.80	5.54										−0.05	0.09
10	S4UJ	2.61	1.11											0.28 *
11	S4UF	5.81	3.92											1

Note: Full items are listed by abbreviations. Growth—Growth creative mindset; Fixed—Fixed creative mindset; AUT—AUT score; S1—Memory retrieval strategy; S2—Splitting strategy; S3—Property-based strategy; S4—General use strategy; UJ—utility judgment; UF—usage frequency. * *p* < 0.05; ** *p* < 0.01.

## Data Availability

The data are currently not publicly available due to participant privacy, but they are available from the first author upon reasonable request.
